# An Ab Initio Study of Connections between Tensorial Elastic Properties and Chemical Bonds in Σ5(210) Grain Boundaries in Ni_3_Si

**DOI:** 10.3390/ma11112263

**Published:** 2018-11-13

**Authors:** Martin Friák, Martin Zelený, Monika Všianská, David Holec, Mojmír Šob

**Affiliations:** 1 Institute of Physics of Materials, Academy of Sciences of the Czech Republic, Žižkova 22, CZ-616 62 Brno, Czech Republic; Monika.Vsianska@seznam.cz (M.V.); mojmir@ipm.cz (M.Š.); 2 Institute of Materials Science and Engineering, NETME Centre, Faculty of Mechanical Engineering, Brno University of Technology, Technická 2896/2, CZ-616 69 Brno, Czech Republic; zeleny@ipm.cz; 3 Faculty of Mathematics and Physics, Charles University in Prague, Ke Karlovu 5, CZ-121 16 Prague, Czech Republic; 4 Central European Institute of Technology, CEITEC MU, Masaryk University, Kamenice 5, CZ-625 00 Brno, Czech Republic; 5 Department of Chemistry, Faculty of Science, Masaryk University, Kotlářská 2, CZ-611 37 Brno, Czech Republic; 6 Department of Physical Metallurgy and Materials Testing, Montanuniversität Leoben, Franz-Josef-Strasse 18, A-8700 Leoben, Austria; david.holec@unileoben.ac.at

**Keywords:** Ni_3_Si, grain boundaries, elasticity, ab initio, stability, phonon, COHP

## Abstract

Using quantum-mechanical methods we calculate and analyze (tensorial) anisotropic elastic properties of the ground-state configurations of interface states associated with Σ5(210) grain boundaries (GBs) in cubic L1${}_{2}$-structure Ni${}_{3}$Si. We assess the mechanical stability of interface states with two different chemical compositions at the studied GB by checking rigorous elasticity-based Born stability criteria. In particular, we show that a GB variant containing both Ni and Si atoms at the interface is unstable with respect to shear deformation (one of the elastic constants, $C_{55}$, is negative). This instability is found for a rectangular-parallelepiped supercell obtained when applying standard coincidence-lattice construction. Our elastic-constant analysis allowed us to identify a shear-deformation mode reducing the energy and, eventually, to obtain mechanically stable ground-state characterized by a shear-deformed parallelepiped supercell. Alternatively, we tested a stabilization of this GB interface state by Al substituents replacing Si atoms at the GB. We further discuss an atomistic origin of this instability in terms of the crystal orbital Hamilton population (COHP) and phonon dispersion calculations. We find that the unstable GB variant shows a very strong interaction between the Si atoms in the GB plane and Ni atoms in the 3rd plane off the GB interface. However, such bond reinforcement results in weakening of interaction between the Ni atoms in the 3rd plane and the Si atoms in the 5th plane making this GB variant mechanically unstable.

## 1. Introduction

Grain boundaries (GBs) represent one of the most important classes of extended defects. Their properties are crucial for many aspects of solid-state materials, including, e.g., their macroscopic strength [1,2,3,4,5]. GBs are becoming even more important due to the recent proliferation of technologies providing and utilizing ultra-fine grained or nano-granular materials. In these materials, the role of GB-affected regions may even dominate when compared with that of the grain interior itself. It also means that suitable design of GB-related states can also possibly lead to material systems with properties significantly different from the bulk [6,7,8,9,10,11,12,13,14,15,16,17,18,19]).

Properties of GBs are very sensitive to compositional changes [20,21,22,23,24,25,26,27,28], which can be, for example, driven thermodynamically. Impurities, even in very low concentrations (ppm), can drastically change GB characteristics (see, for example, Refs. [29,30]). One of the effects, which is usually associated with segregation of impurities on the GB, is intergranular embrittlement. It is accompanied by a profound reduction of the ductility and strength. Materials, which significantly suffer from intergranular fracture and low ductility, are Ni-based Ni${}_{3}$X intermetallic compounds with the L1${}_{2}$ crystal structure [31,32,33] although they exhibit a large potential for high temperature applications in corrosive atmospheres [34,35]. Their cohesive strengths at GB decrease with increasing valence difference between Ni and X atom and with increasing size of X atom in order Ni${}_{3}$Al > Ni${}_{3}$Ga > Ni${}_{3}$Si > Ni${}_{3}$Ge [36,37]. Another explanation derived from behavior of impurities in elemental metals [38] can be based on electronegativity of X atom. The more electronegative atom X at the GB is the higher the tendency for it to pull charge out of the Ni–Ni bonds at the boundary, thereby reducing the cohesive strength and promoting intergranular fracture [37]. In case of Ni${}_{3}$Al, this problem can be solved by adding a small amount of boron, which improves GB cohesion [39,40] and changes intergranular character of fracture to transgranular. The same improvement of properties was, however, not observed after adding boron into Ni${}_{3}$Si intermetallic compound. Boron slightly increases ductility of Ni${}_{3}$Si, while leaving the fracture mode unaffected [31,41]. On the other hand, GB properties can be strongly enhanced by adding a large amount of Ti resulting in a highly ordered Ni${}_{3}$(Si,Ti) alloy [42,43].

The main goal of our present study is to determine and analyze tensorial, in particular elastic, properties of material regions affected by grain boundaries in Ni${}_{3}$Si and to connect interface-induced changes with properties of individual inter-atomic bonds. As far as tensorial elastic properties of GBs are concerned, we build upon the knowledge gained in our recent papers [44,45] which were focused on tensorial characteristics of Σ5(210) grain boundaries in Ni${}_{3}$Al. These studies focused on two possible interface variants, which differ by local chemical composition. Both of them were found to have a significantly reduced shear elastic constant ($C_{55}$) when compared with the bulk. This softening subsequently lowers a homogenized shear modulus and, using a classical approximative model of Slater [46], we predict that it may lead to lowering of the melting temperature [45]. Importantly, Si atoms added close to the GB interface plane of the elastically weaker variant were shown to radically alter the elastic properties and improve the softening. Similar softening leading to the complete mechanical instability of specific Σ5(210) GB in Ni${}_{3}$Si is thoroughly analyzed in the present study together with the stabilization effect of Al impurities. In order to do so, we compute and analyze phonon spectra, crystal orbital Hamilton population and densities of electronic states of the studied interface states.

## 2. Methods

Atomic configurations of GBs are often highly distorted and, therefore, it is truly advantageous to employ reliable theoretical tools, in particular quantum-mechanical (also called ab initio or first-principles) calculations, when studying them [47,48,49,50,51,52,53,54,55]. Our ab initio calculations are based on the density functional theory [56,57] and we used the Vienna Ab initio Simulation Package (VASP) [58,59] including the projector augmented-wave potentials [60]. The electronic wave functions were expanded in plane waves and the expansion was cut-off at those with the kinetic energy of 500 eV. Regarding the exchange and correlation energy, we employed the generalized gradient approximation as parametrized by Perdew, Burke, and Ernzerhof [61]. We used a 5 × 17 × 3 Monkhorst–Pack **k**-point mesh for the 64-atom supercells (15 × 15 × 15 in the case of the 4-atom cell of Ni${}_{3}$Si bulk) and the Methfessel–Paxton smearing method [62] with a 0.1 eV smearing width for integration over the Brillouin zone. To understand the inter-atomic interactions in the studied systems, the crystal orbital Hamilton population (COHP) and density of (electronic) states (DOS) analysis [63] based on projection of plane waves to a local basis [64] implemented in the program LOBSTER 2.1 [65,66] were used.

**Figure 1:**
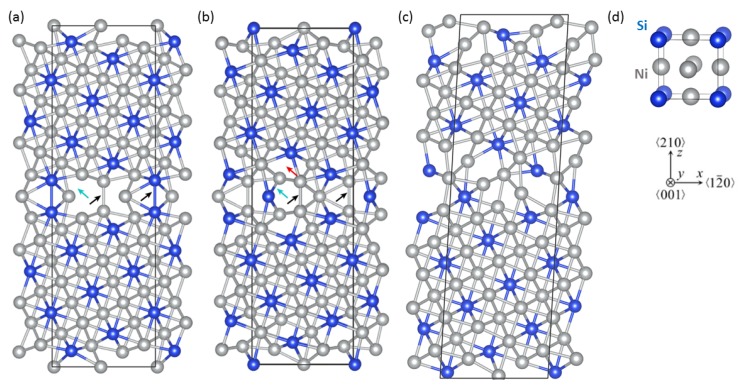
Visualization of computational supercells used in our calculations of interface states related to the Σ5(210) GBs in Ni${}_{3}$Si with different stoichiometries, (**a**) Σ5(210)${}^{\mathrm{Ni},\mathrm{Ni}}$ with only Ni atoms at the GB plane, (**b**) Σ5(210)${}^{\mathrm{Si},\mathrm{Ni}}$ with both Si and Ni atoms at the GB plane (mechanically unstable configuration characterized by a rectangular-parallelepiped supercell), (**c**) mechanically stable shear-deformed state of Σ5(210)${}^{\mathrm{Si},\mathrm{Ni}}$ and (**d**) cubic L1${}_{2}$ unit cell of bulk Ni${}_{3}$Si together with crystallographic directions of GB supercell vectors (before the shear deformation). The Ni atoms are visualized as the blue spheres while the Si atoms as the gray ones. Arrows indicate specific inter-atomic bonds discussed in the text (see below). Please note that some atoms are shown together with their periodic images.

For our study, we have chosen the Σ5(210) GB (see Figure 1) because it is, on one hand, rather symmetric but, on the other hand, it exhibits an additional volume (when compared with the bulk), similarly as it is the case in many non-special GBs. The two different GB chemical compositions corresponding to either solely Ni or both Ni and Si atoms at the GB plane are shown in Figure 1a,b, respectively. It is worth mentioning that the computational supercells are, in fact, periodic approximants of the real GB-associated interface states because the periodic boundary conditions are applied. Our study was aimed at determining thermodynamic, structural and tensorial elastic properties when all atomic positions as well as the shape and the volume of the supercell were fully relaxed reducing the forces on atoms under 1 meV/Å. Finally, when simulating the application of external strains to determine the elastic constants (see Refs. [67,68]), we also relaxed the atomic positions. Our previous results for the same type of the GB in Ni${}_{3}$Al show that the most critical shear elastic constant, $C_{55}$, that could be as low as 15 GPa in case when only Ni atoms are located at the interface of Σ5 (210) in Ni${}_{3}$Al, is much higher (64 GPa) if these atomic relaxations are omitted (see more details in our recent paper [44]).

Lattice-dynamics calculations were performed with the Phonopy [69] package via the supercell finite-displacement method [70]. The second-order force-constants matrices were calculated using 2 × 2 × 1 expansions of the 64-atom supercell describing GBs with a displacement step size of 10${}^{-2}$ Å. The 3 × 3 × 3 expansion of L1${}_{2}$ unit cell was used for the bulk Ni${}_{3}$Si. We found that these cells were sufficiently large to converge the shape of the density of phonon states (DPS). Calculated force-constant matrices were subsequently projected on the unit vector along each bonding direction to obtain information about bond “stiffness” [71]. The DPS curves were constructed by evaluating the phonon frequencies on 25 × 45 × 15 and 20 × 20 × 20 Monkhorst–Pack **q**-point grid for GBs and the bulk, respectively.

## 3. Results

Our quantum-mechanical calculations of the bulk Ni${}_{3}$Si with the L1${}_{2}$ structure predict its lattice parameter to be 3.511 Å in perfect agreement with the experimental value of 3.5002 Å [72]. Regarding Σ5(210) GBs in Ni${}_{3}$Si, the computed GB energies are very similar for both compositional variants, 1.49 and 1.59 J/m${}^{2}$ for the Σ5(210)${}^{\mathrm{Ni},\mathrm{Ni}}$ with only Ni atoms at the interface (Figure 1a) and Σ5(210)${}^{\mathrm{Si},\mathrm{Ni}}$ with both types of atoms (Si and Ni) at the interface (Figure 1b), respectively. The studied GBs have also different volumes when compared with the bulk Ni${}_{3}$Si (see also the lattice parameters in Table 1). This additional volume is an averaged value when the additional volume obtained for the whole computational cell is divided by the total area of the two GBs inside of the supercell (it is thus expressed as a length parameter). Its value is nearly identical for both GB variants, 0.3195 and 0.3189 Å (i.e., Å${}^{3}$/Å${}^{2}$) in the case of the Σ5(210)${}^{\mathrm{Ni},\mathrm{Ni}}$ and Σ5(210)${}^{\mathrm{Si},\mathrm{Ni}}$, respectively. It should be noted that alternative ways of analyzing the additional volume exist in literature (see, e.g., vertical atomic-layer-resolved shifts that are analyzed in the recent study by Kumar et al. [73]).

Next, we determine the elastic properties. The three elastic constants in the case of the cubic Ni${}_{3}$Si at *T* = 0 K amount to $C_{11}$ = 312 GPa, $C_{12}$ = 163 GPa and $C_{44}$ = 130 GPa. They are in reasonable agreement with rather broad range of experimental values ($C_{11}$ is reported to be from 265 GPa [74] to 370 GPa [75], $C_{12}$ from 170 GPa [74] to 212 GPa [76] and $C_{44}$ ranges from 125 GPa [74] to 224 GPa [76]) as well as theoretical values of $C_{11}$ = 269 GPa, $C_{12}$ = 140 GPa and $C_{44}$ = 108 GPa from Ref. [77].

Calculated elastic constants of all studied GB systems are given in Table 2 and Table A1 in the Appendix together with bulk elastic constants in the same coordination system. In addition to providing numerical values of individual elastic constants, we also visualize these elastic properties.

**Figure 2:**
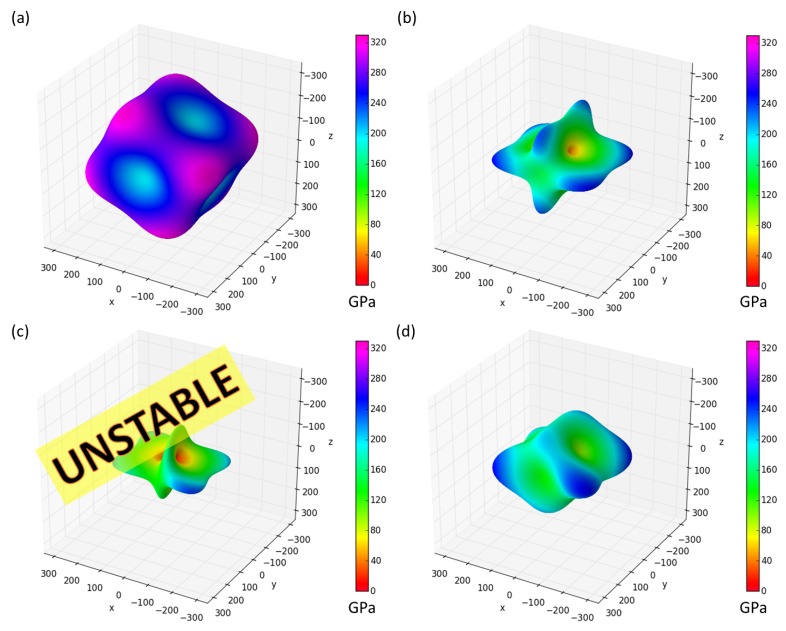
Visualization of directional dependences of the Young’s modulus of the studied Ni${}_{3}$Si systems, (**a**) the Ni${}_{3}$Si bulk in the coordination system of the studied GBs, (**b**) Σ5(210)${}^{\mathrm{Ni},\mathrm{Ni}}$, (**c**) mechanically unstable Σ5(210)${}^{\mathrm{Si},\mathrm{Ni}}$ (rectangular-parallelepiped supercell) and (**d**) mechanically stable Σ5(210)${}^{\mathrm{Si},\mathrm{Ni}}$ (sheared-parallelepiped supercell) when the GBs plane is the *x-y* plane. The directional dependences were visualized by the SC-EMA [80,81,82] library (scema.mpie.de) and derived from the elastic constants (Table 2 and Table A1 in the Appendix).

**Figure 3:**
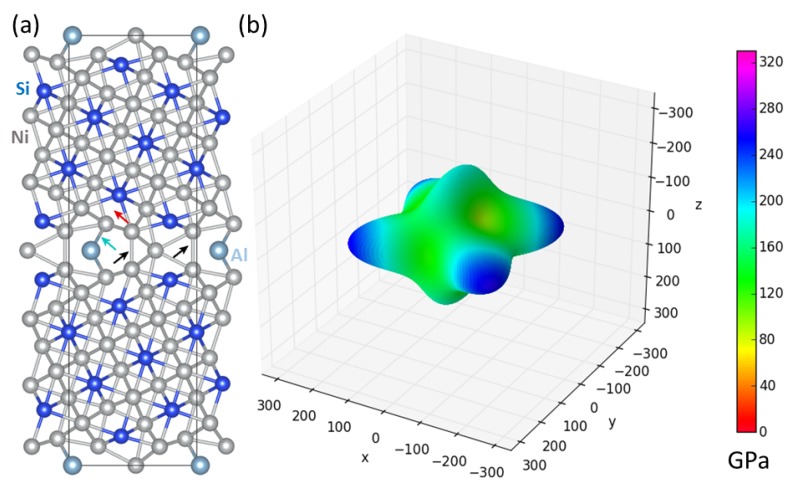
(**a**) Schematic visualization of the supercell of the Σ5(210)${}^{\mathrm{Si},\mathrm{Ni}}$ interface state with Si atoms at the interface plane substituted by Al atoms and (**b**) corresponding directional dependences of the Young’s modulus. The latter was computed and visualized by the SC-EMA [80,81,82] library (scema.mpie.de) based on quantum-mechanically computed elastic constants (Table 2 and Table A1 in the Appendix). Arrows indicate specific inter-atomic bonds discussed in the text (see below).

In particular we show how the studied GB interface states respond to uniaxial loading along different crystallographic directions by exhibiting directional dependences of the Young’s modulus in Figure 2 and Figure 3. Tensorial elastic properties provide the wealth of insight and understanding. For example, they allow us to rigorously assess the mechanical stability of the studied system (employing so-called Born stability criteria [78,79]).

When inspecting Figure 2 and Figure 3, it is obvious that the GB-related interface states Σ5(210)${}^{\mathrm{Ni},\mathrm{Ni}}$ (Figure 2b) and Σ5(210)${}^{\mathrm{Si},\mathrm{Ni}}$ (Figure 2d) exhibit lower maximum values of the Young’s modulus, lower (by ≈ 100 GPa) than the bulk Ni${}_{3}$Si (Figure 2a). The interface states are thus softer than the bulk. Checking the underlying elastic constants in Table 2 in the case of the Σ5(210)${}^{\mathrm{Ni},\mathrm{Ni}}$ compositional variant we see that nearly all elastic constants are lower than those of bulk Ni${}_{3}$Si. The most dramatic reduction is related to $C_{55}$ elastic constant which is equal only to 12 GPa. This is nearly 8 times lower value than in the bulk ($C_{55}$ = 94 GPa). Here, we recall Born stability criteria (see, e.g., [78]) that connect the mechanical stability of a studied system with the positiveness of leading principal minors of the matrix of its elastic constants. The diagonal elements $C_{44}$, $C_{55}$ and $C_{66}$ must be positive and the above-discussed drop predicted for $C_{55}$ thus identifies the weakest link. Also, the elastic anisotropy is significantly enhanced when compared with the bulk. Using the ELATE software [83] the stiffest directions is identified to have the Young’s modulus equal to 257 GPa (color-coded dark blue in Figure 2b), i.e., within the same crystallographic plane as the GB interface, while the softest direction with the Young’s modulus is approaching only 42 GPa (red colors in Figure 2b) and is inclined to the GB interface plane.

The $C_{55}$ elastic constant related to shear deformations has turned out to be crucial for the stability of the studied GB states. In particular, the higher-energy Σ5(210)${}^{\mathrm{Si},\mathrm{Ni}}$ GB described by the original rectangular-parallelepiped atomic configuration (see Figure 1b) is predicted to have the $C_{55}$ elastic constant negative (equal to −98 GPa, directional dependence in Figure 2c is obtained when artificially setting the value of $C_{55}$ to 10 GPa). As this violates the Born stability criteria, this configuration is mechanically unstable. Importantly, our calculations of elastic constants have not only identified the original atomic configuration of Σ5(210)${}^{\mathrm{Si},\mathrm{Ni}}$ GB as mechanically unstable but also allowed us to track the deformation mode, which leads to lowering of the energy when compared to the undeformed state, and find the mechanically stable lowest-energy configuration. It is a sheared atomic configuration (see Figure 1c) and its elastic constants are given in Table 2 and Table A1 mentioned in the Appendix). In particular, the value of $C_{55}$ is predicted to be 37 GPa. This is nevertheless still nearly three times lower than the corresponding bulk value. The Young’s modulus for this configuration is depicted in Figure 2d.

The above-discussed lower value of the shear elastic constant $C_{55}$ is also, in fact, in qualitative agreement with previous findings [84,85] reported for GBs in elemental face-centered cubic (fcc) metals. In these studies, atomistic simulations were combined with a method that allows to decompose an overall elasticity into that of different atomic layers and, similarly as in our study, the shear elastic constants were found reduced close to the GB plane [85].

The overall comparison of the above-discussed cases of Σ5(210)${}^{\mathrm{Ni},\mathrm{Ni}}$ and Σ5(210)${}^{\mathrm{Si},\mathrm{Ni}}$ GB interface states neatly illustrates a very high sensitivity of anisotropic (tensorial) elastic properties of these states to compositional changes at the interface plane. Therefore, we have made a step in the direction of a materials design of GB states and also tried to stabilize the original atomic configuration of Σ5(210)${}^{\mathrm{Si},\mathrm{Ni}}$ GB by substituting the interface Si atom by Al (see atoms in Figure 3a) as a less electronegative element. This substituted state is indeed stable as far as the elastic constants are concerned ($C_{55}$ = 30 GPa). The corresponding elastic constants are listed in Table 2 and Table A1 (in the Appendix) and the directional dependence of the Young’s modulus is exhibited in Figure 3b.

## 4. Discussion

**Figure 4:**
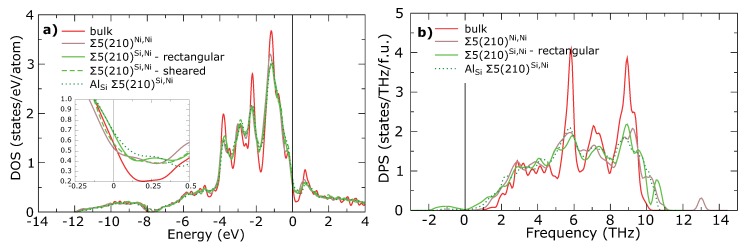
(**a**) Total density of states (DOS) per atom for all studied GBs and bulk Ni${}_{3}$Si. The inset figure shows the DOS around the Fermi level, $E_{F}$, which is set to be the energy zero. (**b**) Total density of phonon states (DPS) per formula unit for all undistorted GBs and bulk Ni${}_{3}$Si (see also the phonon spectra in the Appendix).

For deeper understanding of mechanical and thermodynamic instability of Σ5(210)${}^{\mathrm{Si},\mathrm{Ni}}$ GB and stabilization effect of Al, we further analyze vibrational properties represented by phonons and chemical bonding embodied in electronic structure of studied periodic approximants of GBs. Lattice instabilities could be usually associated with increasing of DOS at the Fermi level, $E_{F}$. Total DOS of all studied GBs are very similar to DOS of bulk Ni${}_{3}$Si (see Figure 4a). Small deviations can be found around the $E_{F}$ (see the inset in Figure 4a). The Σ5(210)${}^{\mathrm{Si},\mathrm{Ni}}$ GB shows significantly higher DOS at the $E_{F}$ than Σ5(210)${}^{\mathrm{Ni},\mathrm{Ni}}$ GB which indicates its lower stability. Decrease of DOS at the $E_{F}$ can be reached by shearing of Σ5(210)${}^{\mathrm{Si},\mathrm{Ni}}$ GB resulting in its stabilization, whereas substitution of Al atoms at the GB leaves DOS almost unchanged.

**Figure A1:**
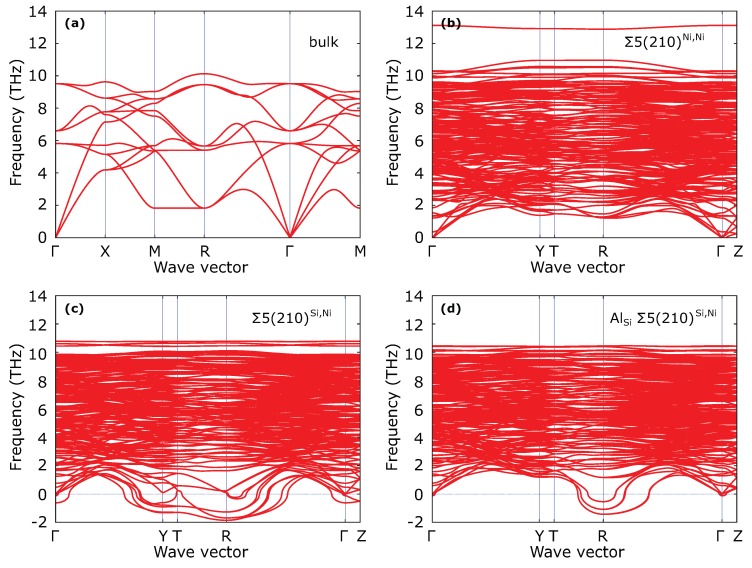
Phonon dispersion relations of (**a**) bulk Ni${}_{3}$Si, (**b**) stable Σ5(210)${}^{\mathrm{Ni},\mathrm{Ni}}$ grain boundary, (**c**) unstable Σ5(210)${}^{\mathrm{Si},\mathrm{Ni}}$ grain boundary and (**d**) Σ5(210)${}^{\mathrm{Si},\mathrm{Ni}}$ grain boundary partially stabilized by substitution of Si at the GB plane by Al.

Stability of GB can be better judged by inspecting the density of phonon states (DPS) in Figure 4b. The Σ5(210)${}^{\mathrm{Si},\mathrm{Ni}}$ GB exhibits significant soft modes with maxima of negative frequencies in phonon dispersion relation (not shown) for **q** = (0, 0.5, 0) and **q** = (0.5, 0.5, 0.5), see Figure A1 in the Appendix. These soft modes are not presented in DPS curve of stable Σ5(210)${}^{\mathrm{Ni},\mathrm{Ni}}$ GB, which, on the other hand, contains a high-frequency peak at 13 THz corresponding to Si atoms in the 2nd layer of the GB interface. Imaginary part of DPS is significantly reduced for Σ5(210)${}^{\mathrm{Si},\mathrm{Ni}}$ GB with substituted Al atom (see Figure A1 in the Appendix). The soft mode at **q** = (0, 0.5, 0) disappears after substituting of Si by Al, but the soft mode at **q** = (0.5, 0.5, 0.5) still preserves. This indicates that Σ5(210)${}^{\mathrm{Si},\mathrm{Ni}}$ GB is not fully stabilized by Al.

**Figure 5:**
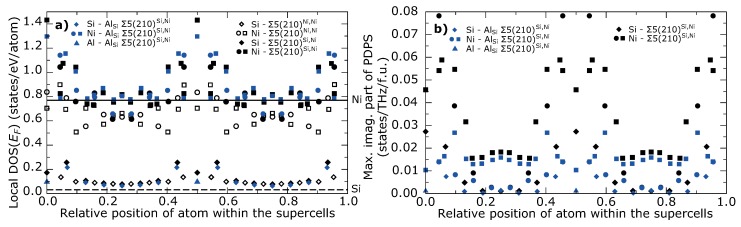
(**a**) Local density of states (DOS) at the Fermi level, $E_{F}$, for each atom as a function *z*-coordinate in the supercell in all types of Σ5(210) GB. Horizontal lines represents local DOS at the $E_{F}$ in bulk Ni${}_{3}$Si. (**b**) The highest partial density of phonon states (PDPS) in imaginary frequencies for each atom as a function of *z*-coordinate in the supercell in both Σ5(210)${}^{\mathrm{Si},\mathrm{Ni}}$ GB with and without substituted Al atom. Squares represent Ni atoms in the layer occupied only by Ni atoms and circles represent Ni atoms in the layer occupied by Ni and Si atoms.

To identify which particular atoms contribute to instability of GBs, we further plotted values of local DOS at the $E_{F}$ as a function of *z*-coordinates in the supercell in Figure 5a.

Both Σ5(210)${}^{\mathrm{Si},\mathrm{Ni}}$ GB with and without substituted Al atom exhibit increased DOS at $E_{F}$ for atoms close to GB region when compared with the bulk Ni${}_{3}$Si. The highest DOS at the $E_{F}$ can be found for Ni atom exactly at the GB plane in Σ5(210)${}^{\mathrm{Si},\mathrm{Ni}}$ GB without Al atom. Substitution of Al atom results in a decrease of this value, which could indicate stabilization of GB. However, DOS at the $E_{F}$ for Ni atoms in the 2nd layer slightly increases. The stable Σ5(210)${}^{\mathrm{Ni},\mathrm{Ni}}$ GB does not show this behavior and the DOS value at the $E_{F}$ are very close to bulk values also for atoms at the GB region.

The significance of the stabilization effect of Al at GB may be seen from Figure 5b where we plot the highest partial DPS for imaginary frequencies for both Σ5(210)${}^{\mathrm{Si},\mathrm{Ni}}$ GB. The highest contribution to soft modes for GB without Al arise from Ni atoms in the 2nd layer. Also contributions of atoms in the 3rd and 4th layer are high. Surprisingly, Ni atom at GB does contribute so significantly and contribution of Si atom at GB is just slightly higher than contribution of other Si atoms in the supercell. Substitution of Si by Al significantly decreases imaginary phonon states for all atoms, with the strongest effect for atoms at the GB plane. The highest contribution to imaginary modes now can be found for atoms in the 4th layer. This analysis of imaginary phonon states shows that instability of Σ5(210)${}^{\mathrm{Si},\mathrm{Ni}}$ GB arises from atoms in the neighborhood of the GB plane than from atoms exactly at the GB plane.

To get a deeper insight into mutual chemical interaction between individual atoms we employed the analysis of crystal orbital Hamilton population (COHP) [63], which helps us to identify weaker chemical bonds in studied periodic approximants of GBs. Here a negative value of COHP represents bonding interaction in particular bands, whereas a positive COHP corresponds to antibonding interactions. Bands which do not participate in bonding between particular atoms do not appear in COHP curves. Thus, an integral of COHP up to Fermi level (ICOHP) represents the “chemical strength” of interaction between two atoms, where more negative value of ICOHP means a stronger interaction [86].

Another way how to judge the strength of a chemical bond from the point of view of lattice dynamics is a projection of the force constant obtained from phonon calculation on the unit vector along each bonding direction. It provides information about “bond stiffness” for a particular pair of atoms [71]. In the bulk Ni${}_{3}$Si (see Table 3), both nearest neighbor interactions exhibit almost the same ICOHP, but their bond-projected force constants, $\varphi_{B}$, differ. The Ni–Ni bond seems to be much stronger than Ni–Si bonds, which is in disagreement with previous analysis of bonds in Ni${}_{3}$Si based on charge-density plots [87,88]. Next-nearest neighbor interactions exhibit significantly smaller values of both quantities and probably have just a side effect on the stability of Ni${}_{3}$Si lattice.

**Figure 6:**
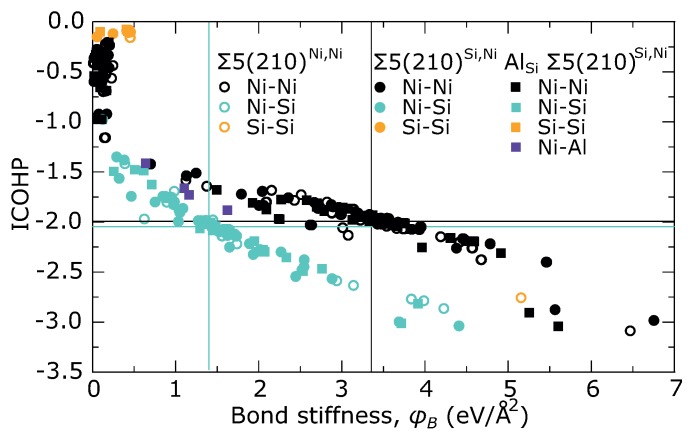
Integral of COHP up to the Fermi level as a function of projected force constant $\varphi_{B}$ (bond stiffness) for Σ5(210)${}^{\mathrm{Ni},\mathrm{Ni}}$ GB (open circles), Σ5(210)${}^{\mathrm{Si},\mathrm{Ni}}$ GB (full circles) and Σ5(210)${}^{\mathrm{Si},\mathrm{Ni}}$ GB with Al (full squares). Black solid lines correspond to values for nearest neighbors Ni–Ni bond in bulk Ni${}_{3}$Si, whereas cyan solid lines represent Ni–Si bond.

Figure 6 shows the ICOHP values as a function of the projected force constant $\varphi_{B}$ for all studied GBs. Several regions can be recognized in each trend. The weakest interactions between the next nearest neighbors are located in the upper-left corner of the each respective trend.

**Figure 7:**
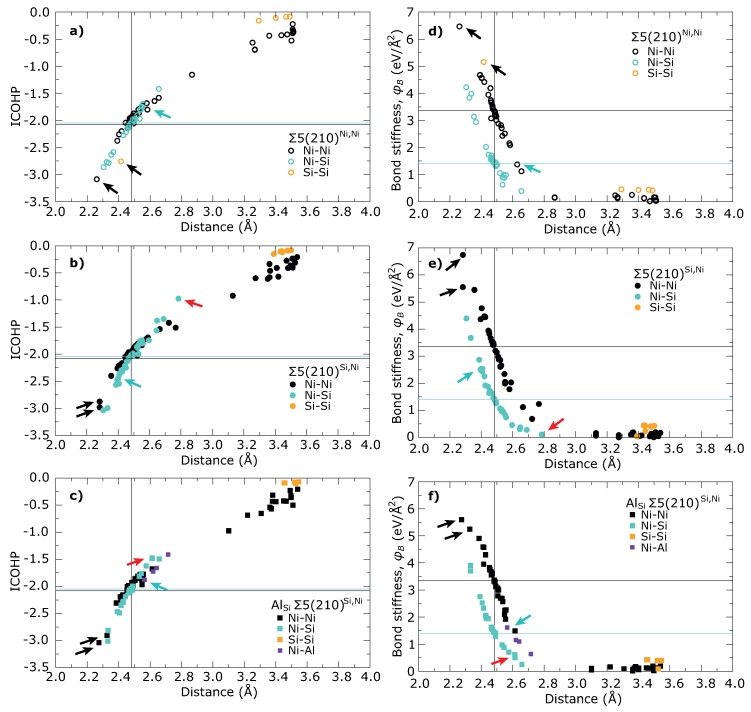
Left column: Integral of COHP up to the Fermi level as a function of the bond length for (**a**) Σ5(210)${}^{\mathrm{Ni},\mathrm{Ni}}$ GB, (**b**) Σ5(210)${}^{\mathrm{Si},\mathrm{Ni}}$ GB and (**c**) Σ5(210)${}^{\mathrm{Si},\mathrm{Ni}}$ GB with Al. Black solid lines correspond to values for nearest neighbors Ni–Ni bond in the bulk Ni${}_{3}$Si, whereas cyan solid lines represent Ni–Si bond. Right column: Projected force constant $\varphi_{B}$ (bond stiffness) as a function of the bond length for (**d**) Σ5(210)${}^{\mathrm{Ni},\mathrm{Ni}}$ GB, (**e**) Σ5(210)${}^{\mathrm{Si},\mathrm{Ni}}$ GB and (**f**) Σ5(210)${}^{\mathrm{Si},\mathrm{Ni}}$ GB with Al. Black solid lines correspond to values for nearest neighbors Ni–Ni bond in bulk Ni${}_{3}$Si, whereas cyan solid lines represent Ni–Si bond. Arrows mark inter-atomic bonds discussed in text.

The strongest interaction can be found in the bottom-right corner, which corresponds to interactions across the GB plane between the atoms from the 2nd layers. In case of Σ5(210)${}^{\mathrm{Si},\mathrm{Ni}}$ GBs, there are two Ni–Ni interactions in the supercell, whereas the Σ5(210)${}^{\mathrm{Ni},\mathrm{Ni}}$ GB contains only a single Ni–Ni interaction and a single Si–Si interaction of this type. These interactions are marked by black arrows in Figure 1 as well as in Figure 7, which show ICOHP and $\varphi_{B}$ as functions of bond length, respectively.

**Figure 8:**
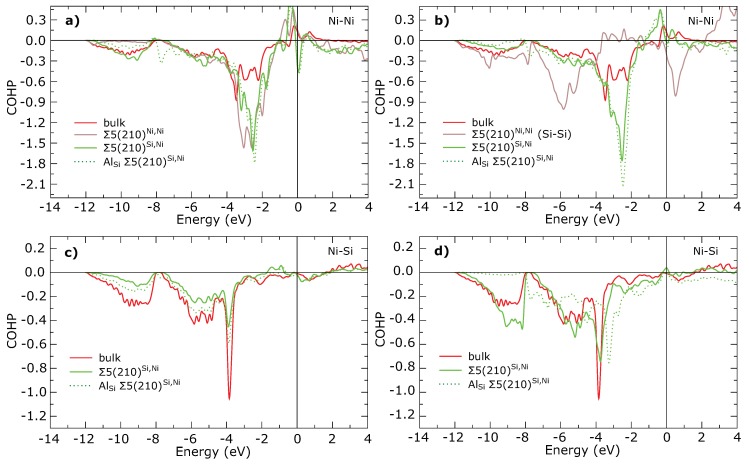
COHP curves for selected interatomic interactions in GBs and corresponding interactions in the bulk. (**a**) Interaction across the GB of Ni–Ni atoms from Ni plane between both 2nd layers (marked by a black arrow in other figures). (**b**) Interaction across the GB of Ni–Ni (Si–Si) atoms from NiSi plane between both 2nd layers (marked by black arrows in other figures). (**c**) Interaction of Ni–Si atoms between the 3rd and 5th layer (marked by red arrows in other figures). (**d**) Interaction of Ni–Si(Al) atoms between the GB and the 3rd layer (marked by cyan arrows in other figures).

Full COHP curves for these interactions are plotted in Figure 8a,b together with Ni–Ni interaction in the bulk Ni${}_{3}$Si. Enhancement of d-d bonding band around 3 eV below the $E_{F}$ is significant for all Ni–Ni bonds across the GB compared with Ni–Ni interaction in the bulk Ni${}_{3}$Si. This enhancement is not affected by the substitution by the Al atoms. The Si–Si interaction in stable Σ5(210)${}^{\mathrm{Ni},\mathrm{Ni}}$ GB (orange open circles in Figure 6 and Figure 7) seems to be weaker than Ni–Ni interaction (see Figure 8b). This conclusion is in line with the values of $C_{33}$ elastic constants in Table 2 which are lower for the studied GB states than in the bulk but still high enough to guarantee the mechanical stability.

Figure 6 further contains two almost linear dependencies for Ni–Ni and Ni–Si nearest neighbor interactions, which show that ICOHP is very well correlated with $\varphi_{B}$ for a particular type of bond. As can be seen from Figure 7 the Σ5(210)${}^{\mathrm{Si},\mathrm{Ni}}$ GB contains also a large number of the Ni–Si bonds, which are significantly stronger than Ni–Si bonds in the bulk. The Ni-Al bonds in the substituted Σ5(210)${}^{\mathrm{Si},\mathrm{Ni}}$ GB (purple squares) exhibit a character more similar to Ni–Ni bonds than to Ni–Si bonds although Al replaces Si. To explain the instability of Σ5(210)${}^{\mathrm{Si},\mathrm{Ni}}$ GB we are looking for the weakest nearest neighbors interactions. It can be found between the Ni atoms in the 3rd layer and the Si atoms in the 5th layer (marked by red arrows in Figure 1 and Figure 7) and shows also the longest inter-atomic distance out of all Ni–Si bonds, significantly longer than the Ni–Si bond in the bulk. Strengthening of this interaction as well as shortening of the bond length can be seen after replacing the Si atom at the GB plane by an Al atom. Although strengthening does not reach the level found in the case of the Ni–Si interaction in bulk, it is sufficient to stabilize the GB. A comparing of COHP curves in Figure 8c shows that the character of interactions in both GBs is very similar to that in the bulk and it is just suppressed or enhanced due to a shorter or longer bond length.

To find the explanation for the weakening of this bond, we have to look closer at the GB plane. In particular, interaction between the Si atoms in the GB plane and the Ni atoms in the 3rd layer (marked by cyan arrows in Figure 1 and Figure 7) belongs to that Ni–Si interaction with enhanced strength. As can be seen in Figure 8d, this enhancement arises from a strong s–s interaction at the bottom of valence bands. This very strong interaction, on the other hand, results in a weakening of the previously discussed Ni–Si bond between the 3rd and the 5th layer. When the Si atom in the GB plane is replaced by the less electronegative Al atom, the corresponding Ni-Al bond exhibits a significantly lower strength due to the missing s–s interaction. On the other hand this weak interaction allows a tighter Ni–Si binding between the 3rd and the 5th layers which stabilizes the Σ5(210)${}^{\mathrm{Si},\mathrm{Ni}}$ GB. Thus, we can conclude that the Σ5(210)${}^{\mathrm{Si},\mathrm{Ni}}$ GB is not unstable due to a weak interaction between some atoms, but due to a strong interaction between the Si atoms in the GB plane and Ni atoms in the 3rd plane.

**Figure 9:**
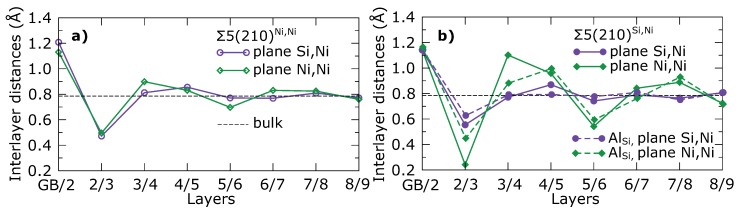
*Ab initio* computed distances between the (210) atomic planes in the supercells modeling (**a**) Σ5(210)${}^{\mathrm{Ni},\mathrm{Ni}}$ and (**b**) Σ5(210)${}^{\mathrm{Si},\mathrm{Ni}}$ interface states without (solid line) and with (dashed line) substituted Al atom at GB compared with the value calculated for the bulk.

Stronger bonds between the Si atoms at the GB plane and Ni atoms in the 3rd layer can be also recognized from inter-layer distances shown in Figure 9. The value of the distance between the 2nd and 3rd layer (marked as ”2/3” in Figure 9) is, in the case of atomic planes containing only Ni atoms in the Σ5(210)${}^{\mathrm{Si},\mathrm{Ni}}$ GB, significantly shorter than all the other inter-planar distances between the 2nd and 3rd layers. Similarly, the distance between the 3rd and 4th layer in the same plane is significantly larger than all the other inter-planar distances between the 3rd and 4th layers. This indicates that the Ni atoms in the 3rd layer are strongly pulled towards the Si atoms at the GB plane. Inter-layer distances in Figure 9b also show that the Ni atoms in the 3rd layer are pushed back to the position far from the GB plane, when the Si atom is replaced by the Al atom.

## 5. Conclusions

We have performed an ab initio study of tensorial elastic properties of the interface states in Ni${}_{3}$Si associated with the Σ5(210) grain boundary. Rather complex tensorial elasto-chemical aspects of the studied periodic GB approximants were shown in the case of states with different atoms at the GB plane (either only Ni atoms (Σ5(210)${}^{\mathrm{Ni},\mathrm{Ni}}$) or both Si and Ni atoms (Σ5(210)${}^{\mathrm{Si},\mathrm{Ni}}$)). The Σ5(210)${}^{\mathrm{Ni},\mathrm{Ni}}$ GB state is predicted to have only a slightly lower interface energy of the two studied variants. The elastic constants are found to depend very sensitively on the GB plane chemical composition. In particular, the GB variant containing both Ni and Si atoms at the interface is shown to be unstable with respect to a shear deformation (one of the elastic constants, $C_{55}$, is negative). This instability is found for a rectangular-parallelepiped supercell obtained when applying a standard coincidence-lattice construction. Our elastic-constant analysis allowed us to identify a shear-deformation mode reducing the energy and eventually to obtain a mechanically stable ground-state characterized by a shear-deformed parallelepiped supercell. Nevertheless, a three-/eight-fold reduction of the elastic constant $C_{55}$ (when compared with the bulk value) is identified as the crucial weakest link for the mechanical stability for the studied GB states. We have also partly stabilized this GB interface state by Al substituent replacing Si atoms at the GB.

Next, an origin of this elastic softening and instability of the rectangular-parallelepiped Σ5(210)${}^{\mathrm{Si},\mathrm{Ni}}$ GB variant is discussed in terms chemical inter-atomic interactions described by the crystal orbital Hamilton population (COHP). Lattice-dynamics properties represented by projected force-constant matrices on the unit vector along each bonding direction were considered as well. Such complex analysis reveals a weak interaction far from the GB interface between the Ni atoms in the 3rd plane and the Si atoms in the 5th plane. However, this bond weakening is a consequence of a very strong interaction between the Si atoms in the GB plane and Ni atoms in the 3rd plane off the GB interface. The same strong interaction was not observed when Si atom at the GB is replaced by Al. Thus the strong interaction near the GB plane makes this GB variant mechanically unstable.

Our study thus demonstrates very clearly shows the importance of anisotropic elastic-constant analysis for next studies of interface states close to GBs when determining their mechanical (in-)stability. Our analysis represents a complement to numerous previous studies of GBs which were focused predominantly on scalar characteristics (i.e., energy, strength, changes in inter-atomic bonds, as well as electronic structure and atomic configuration, see e.g., Refs. [4,20,89,90,91,92,93,94,95,96,97,98,99,100,101]). Our small-deformation anisotropic-elasticity assessment should be ideally extended in future by simulations of larger deformations [102,103,104,105] which have been rather rarely studied so far in case of GBs (see, e.g., Ref. [106]). The found sensitivity of the elasto-chemical inter-relations, which was additionally exemplified by studying the impact caused by Al atoms substituting Si atoms at the GB interface plane, paves a new approach towards a solute-controlled design of interface states with on-demand tensorial elastic properties and stability.

## Figures and Tables

**Table 1:** The calculated lattice parameters within the (210) interface plane of the studied GBs. The values are compared with those obtained for the bulk for the same plane. The lattice parameters along the $\langle 1\bar{2}0\rangle$ direction, *x*, and 〈001〉 direction, *y* (see Figure 1) are included together with their changes (expressed relatively in % with respect to the bulk).

	Bulk	$\Sigma 5{\left(210\right)}^{\mathrm{Si},\mathrm{Ni}}$		$\Sigma 5{\left(210\right)}^{\mathrm{Ni},\mathrm{Ni}}$	
	**(Å)**	**(Å)**	**(%)**	**(Å)**	**(%)**
*x* $\langle 1\bar{2}0\rangle$	7.853	7.735	−1.49	7.808	−0.56
*y* 〈001〉	3.512	3.523	0.31	3.515	0.09

**Table 2:** The computed elastic constants (all in GPa) of (i) the Ni${}_{3}$Si bulk in the coordination system of the studied GBs (shown in Figure 1), (ii) two types of Ni${}_{3}$Si Σ5(210) GBs with different atoms (see Figure 1) at the interface and (iii) systems with Al atom substituting Si atom within the GB interface plane Ni${}_{3}$(Si,Al${}_{\mathrm{Si}}$) Σ5(210)${}^{\mathrm{Si},\mathrm{Ni}}$. In the case of Σ5(210)${}^{\mathrm{Si},\mathrm{Ni}}$ we list elastic constants for the rectangular-parallelepiped supercells (these systems are mechanically not stable) as well as the shear-deformed ones (these are mechanically stable).

Ni_3_Si States:	$C_{11}$	$C_{12}$	$C_{13}$	$C_{22}$	$C_{23}$	$C_{33}$	$C_{44}$	$C_{55}$	$C_{66}$
bulk 〈1$\bar{2}$0〉 〈001〉 〈210〉	346	164	128	310	164	348	130	94	130
Σ5(210)${}^{\mathrm{Ni},\mathrm{Ni}}$	301	155	135	274	165	276	96	12	115
Σ5(210)${}^{\mathrm{Si},\mathrm{Ni}}$ rectangular	277	163	151	265	168	259	51	−98	108
Σ5(210)${}^{\mathrm{Si},\mathrm{Ni}}$ sheared	387	166	136	264	162	285	79	37	114
Al${}_{\mathrm{Si}}$ Σ5(210)${}^{\mathrm{Si},\mathrm{Ni}}$	282	172	131	256	152	283	61	30	115

**Table 3:** Properties of nearest and next nearest neighbor bonds in bulk Ni${}_{3}$Si: bond length *d*, projected force constant $\varphi_{B}$ and integral of COHP up to the Fermi level.

	d (Å)	$\varphi_{B}$ (eV/Å${}^{2}$)	ICOHP
Ni–Ni	2.4828	3.35	−2.08
Ni–Si	2.4828	1.40	−2.05
Si–Si	3.5113	0.46	−0.07
Ni–Ni (Ni–Si plane)	3.5113	0.19	−0.60
Ni–Ni (Ni plane)	3.5113	0.03	−0.41

## Appendix A

**Table A1:** Additional elastic constants (all in GPa) for the systems which are unstable, sheared or, in the case of the Ni${}_{3}$Si bulk, rotated into a coordination system in which the number of non-zero elastic constants is not minimum (unit cell vectors do not match the axes of the Cartesian coordination system).

Ni_3_Si States:	$C_{14}$	$C_{15}$	$C_{16}$	$C_{24}$	$C_{25}$	$C_{26}$	$C_{34}$	$C_{35}$	$C_{36}$	$C_{45}$	$C_{46}$	$C_{56}$
bulk 〈1$\bar{2}$0〉 〈001〉 〈210〉	0	26.5	0	0	0	0	0	−26.5	1	0	0	0
Σ5(210)^Si,Ni^ rectangular	0	−3	0	1	13	−3	1	16	7	−12	−1	−4
Σ5(210)^Si,Ni^ sheared	0	−5	0	−1	6	1	2	−4	0	1	9	−2
